# Breaking the silence: AI’s contribution to detecting vertebral fractures in opportunistic CT scans in the elderly—a validation study

**DOI:** 10.1007/s11657-025-01524-5

**Published:** 2025-03-25

**Authors:** Anna Spångeus, Tomas Bjerner, Maria Lindblom, Christoph Götz, Allan Hummer, Christoph Salzlechner, Mischa Woisetschläger

**Affiliations:** 1 Division of Diagnostics and Specialist Medicine, Department of Health, Medicine and Caring Sciences, Linköping University, Linköping, Sweden; 2 Department of Activity and Health, Linköping University Hospital, Linköping, Sweden; 3 Center for Medical Image Science and Visualization (CMIV), Linköping, Sweden; 4 Department of Radiology, Linköping University Hospital, Linköping, Sweden; 5 Department for Research and Development, IB Lab, Vienna, Austria; 6 Department of Acute Internal Medicine and Geriatrics and Department of Medical and Health Sciences, Linköping University, Campus US, 581 83 Linköping, Sweden

**Keywords:** Osteoporosis, Vertebral fracture, Artificial intelligence, Opportunistic screening, Computer tomography, Geriatric

## Abstract

**Summary:**

Vertebral fractures frequently go undetected in clinical practice. AI-assisted detection on CT scans demonstrates considerable promise, with a sensitivity of 86% and a specificity of 99%. The performance varied based on sex, and CT kernel, showing superior results in females and in scans using non-bone kernel protocols.

**Purpose:**

Vertebral fractures (VFs) are highly underdiagnosed, necessitating the development of new identification methods for opportunistic screening in computed tomography (CT) scans. This study validated an AI algorithm (ImageBiopsy Lab [IBL], FLAMINGO) for detecting VFs in a geriatric cohort, with various subgroup analyses including different CT protocols.

**Methods:**

The performance of the AI in detecting VFs was compared to assessments by two experienced radiologists. A total of 246 thoracic or abdominal CT scans, primarily conducted for purposes other than skeletal examination, were included in the study.

**Results:**

The patients had a mean age of 84 years (range 62 to 103), with 42% being female. The AI demonstrated high accuracy (0.93), sensitivity (0.86), and specificity (0.99) in detecting moderate to severe VFs. Subgroup analysis revealed accuracy ranging from 0.88 to 0.96, with higher accuracy in females compared to males (0.96 vs. 0.89, *p* = 0.03) and in scans performed with non-bone kernel versus bone kernel protocols (0.96 vs. 0.88, *p* = 0.02). No significant differences were found for age, contrast phase, or spinal region.

**Conclusion:**

The results indicate that the AI algorithm exhibits high performance in a geriatric setting. If effectively integrated with a fracture liaison service, this could enhance VF detection considerable in the future.

## Introduction

Fragility fractures are associated with deterioration of bone micro-architecture and a reduction in bone mass. Although etiology varies, age has a strong contribution to risk of developing osteoporosis and suffering from osteoporotic fractures [1].

Especially vertebral fractures represent a significant health concern due to significant morbidity and mortality and a high risk of subsequent fractures [2–4]. Effective treatments are currently available that significantly reduce the risk of subsequent fractures and mortality [5–9]. Paradoxically, despite their clinical significance and existence of effective preventive treatments, less than one-third of vertebral fractures are diagnosed today [10–12]. This diagnostic gap highlights the need for improved identification methods to facilitate early intervention and prevent future fractures. One potential avenue for achieving this objective involves increasing the reporting of vertebral fractures during radiological examinations. This should also include computed tomography (CT) scans conducted for reasons unrelated to bone health assessment [13].

Artificial intelligence (AI) algorithms have emerged as a promising tool for improving the detection of vertebral fractures in an opportunistic context using CT scans [14–18]. As a screening tool, it is important to consider the risk of over- and/or under-diagnosis.

Although promising, several previous studies conducted on middle-aged cohorts (mean ages 62–73 years and over 50 years, respectively) have demonstrated moderate specificity or sensitivity below 0.7, potentially limiting their clinical applicability [14, 16, 17]. In a previous study, Nicolaes et al. [18] reported promising results with high performance including AUC of up to 0.88, in training and validation data set. The algorithm was later included in the recently MDR cleared IB Lab FLAMINGO (IB Lab GmbH, Vienna, Austria).

To effectively translate the advancements of AI supported vertebral fracture detection into clinical practice, it is important to evaluate the algorithms in real-world settings, including various radiological CT protocols and patient characteristics. Such assessments should consider various imaging parameters and patient characteristics that might influence the algorithm’s performance. These parameters may for example include the presence of contrast agents or not, kernel settings, and different anatomic sites.

In the present study, we aimed to investigate the performance of the IB Lab FLAMINGO algorithm using a real-world dataset obtained during routine clinical practice. This dataset encompasses a diverse range of clinical questions and CT protocols. Our objectives were twofold: (1) to assess performance in real-world opportunistic data from a geriatric cohort, and (2) to explore whether factors such as age, sex, contrast phase, anatomic site, and kernel setting influence the algorithm’s performance.

## Material and methods

### Dataset

**Fig. 1:**
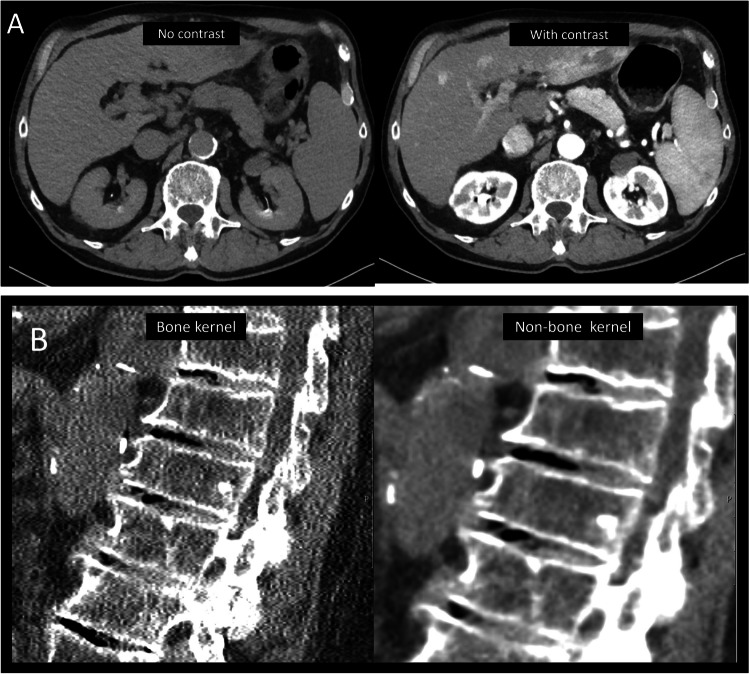
Images showing examples of different contrast phases and kernels. **A** Datasets without (left) and with (right) contrast agent visualizing the uptake of contrast agent in the aorta, kidneys, liver, pancreas and spleen, and to a lesser extend in the trabecular portion of the vertebrae. **B** Datasets with bone kernel (left) and non-bone kernel (right) illustrating the differences in image noise and edge sharpness

The present validation study was based on existing CT scans that were primarily conducted for purposes other than vertebral assessment, yet they included the thoracic and/or lumbar spine. The scans were acquired using various contrast phases and imaging parameters (e.g., kernel) (Fig. 1A and B). All images had a slice thickness of 1 mm.

### Patient selection criteria

All patients identified were previously included in another study focused on falls and fall prevention during in-hospital care in the geriatric ward of the Linköping University Hospital during the period 2018–2020 [19]. The Picture Archiving and Communication System (PACS) was queried to determine whether these patients had undergone a CT scan of thorax and/or abdomen for any reason within 6 months before or after the fall event. The cohort consisted of both females and males, and age ranged from 62 to 103 years. Patient characteristics, including ICD-coded diagnosis and current medication, were obtained from the digital case records. Current medical treatments were defined as ongoing prescriptions at the time of the fall (within 24 h before the fall), except for zoledronic acid, which was considered a current treatment if administered within a year before the fall incident.

### Image acquisition

The clinical CT images were obtained for diverse clinical reasons, including CT scans for thoracic pathologies (e.g., pneumonia, pulmonary embolism, and pleural effusion), aortic assessment, spinal imaging, and abdominal pathologies (e.g., kidney stones, bowel examination, and tumor screening). These scans could be performed with or without contrast enhancement. The scanners utilized were various Siemens models (SOMATOM Force, SOMATOM Drive, SOMATOM Edge, SOMATOM Definition AS + ; Siemens Healthineers, Erlangen, Germany) as well as PET/CT scans from a GE machine (Discovery 710, GE Healthcare, Chicago, USA).

### Ground truth assessment

Each scan underwent evaluation by two experienced radiologists, who independently reviewed the images without knowledge of each other’s assessments. The thoracal datasets were assessed by MW and TB, with 16 and 24 years of radiology experience, respectively, and the abdominal cases were assessed by MW and ML with 16 and 20 years of radiology experience, respectively. In cases of disagreement, a consensus decision was reached. The vertebrae were semi-quantitatively graded according to Genant’s score, ranging from 0 to 3 [20]. Specifically, our study focused on moderate to severe vertebral fractures (grade 2–3). Thus, in the analysis, patients were classified as having a moderate or severe vertebral fracture (grade 2–3) or not (grade 0–1), based on the worst result in each scan.

#### Artificial intelligence analysis

AI analysis was performed using IB Lab FLAMINGO software (IB Lab GmbH, Vienna, Austria). IB Lab FLAMINGO is an automated vertebral fracture detection software that leverages two AI components: (1) a vertebra identification model that pinpoints each vertebra fully visible within the scan; and (2) a vertebral fracture detection model ensemble that assesses fracture severity on the vertebra level. The software processes CT scans of the thoracal and lumbar region and produces estimated Genant fracture grades for each identified vertebra. Cervical vertebral fractures are not analyzed.

The vertebral fracture detection ensemble consists of four individual models that were developed and published previously by Nicolaes et al. [21]. The algorithm uses an open-source vertebra identification model with weights trained on the Verse2020 challenge data available at https://hub.docker.com/r/christianpayer/verse20.

**Fig. 2:**
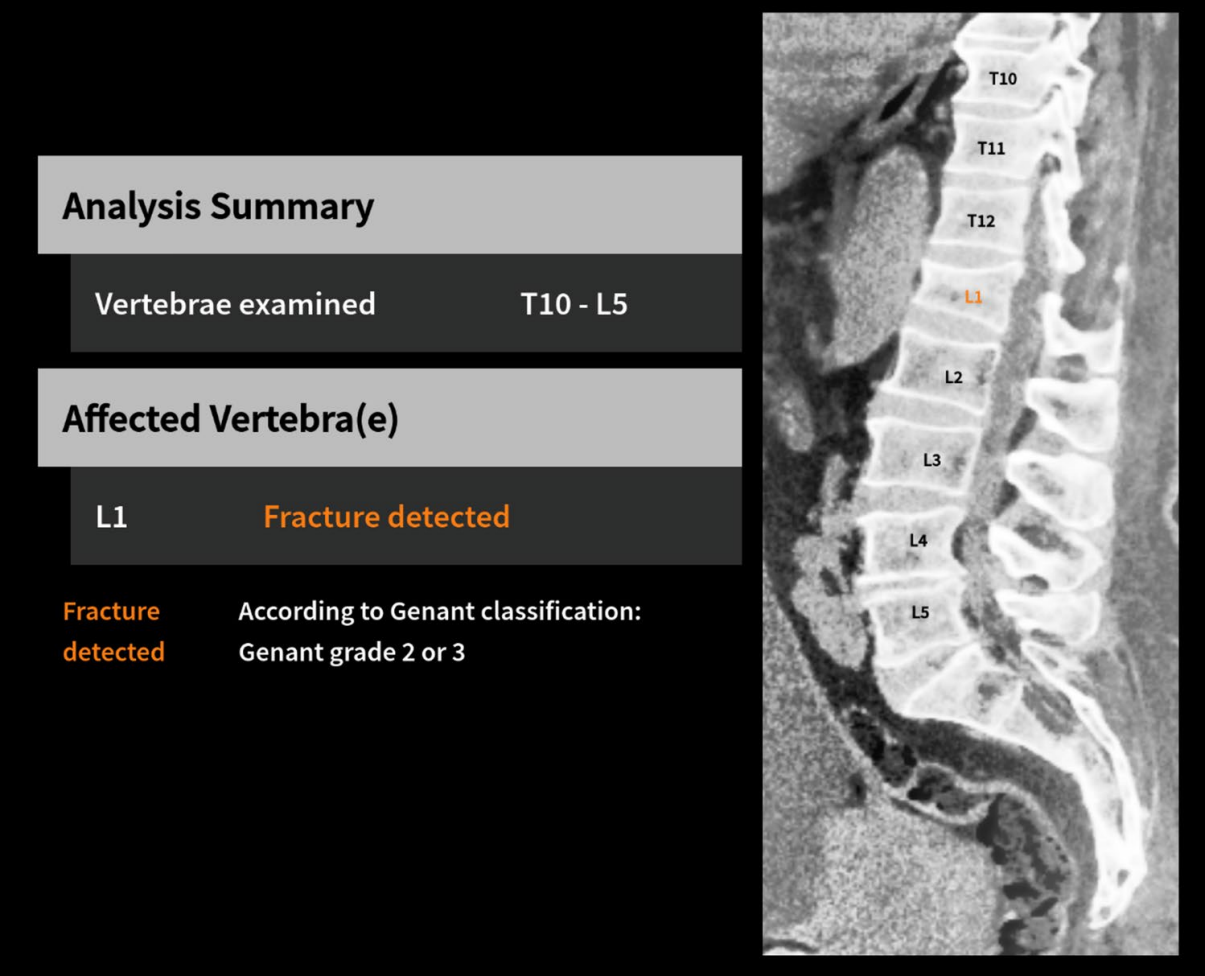
Result from IB Lab FLAMINGO illustrating which vertebrae were analyzed (here T10–L5) and which vertebrae were found fractured (here L1). Only vertebral fractures with a Genant grade of 2 or 3 were reported

The two AI components were packaged as a Docker (docker.com) image with a medical-grade software system that takes care of pre- and post-processing, result aggregation, and reporting as well as communication with the host environment. The software system was programmed in Java 17 (Oracle, Austin, USA). An example image of the result image is given in Fig. 2.

Like ground truth assessment, the IB Lab FLAMINGO software reported the presence of vertebral fractures when moderate or severe vertebral fractures (Genant grade 2–3) were detected and no vertebral fracture when no or mild vertebral fractures (Genant grade 0–1) were detected for each vertebra in the scan. Subject analyses were based on the worst result in each scan.

### Statistics

Statistical analyses were performed in SPSS (version 29.0.1.1, IBM) and Python version 3.9 with the scikit package. Continuous variables were reported as mean and standard deviation. Categorical parameters were reported as numbers or percentages. Statistical analysis comparing groups was performed using Student’s *t*-test for continuous variables and Chi square test for categorical variables and subgroup analysis of accuracy. A *p*-value < 0.05 was considered significant.

## Results

### Descriptive

A total of 101 patients were included in this study, with a mean age of 84 ± 7 years (range 62 to 103 years). Among these patients, 42 were females, and 59 were males. We analyzed a total of 246 scans (1 to 5 scans per patient), with 128 scans covering the thoracic spine (eventually containing the upper lumbar spine) and 118 scans covering the lumbar spine (eventually containing the lower thoracic spine). In these images, we meticulously assessed 2136 vertebrae. For each individual scan, we considered the vertebral fracture with the highest Genant Grade for further analysis.

Most of the scans, i.e., 138 (56%), were conducted without contrast enhancement, while 108 scans were performed with contrast agent (44%). Forty-one percent (*n* = 100) of the CT scans utilized a bone kernel, whereas the remaining scans employed non-bone kernels.

Moderate or severe vertebral fractures were detected in 111 of 246 (45%) scans. Of the 111 scans in which vertebral fractures were detected, 44% (*n* = 49) were reported in the original CT radiology report. Patients with moderate/severe vertebral fractures more frequently exhibited a history of previous fractures (any fracture), ICD-coded vertebral fractures, diagnosed osteoporosis, and received treatment for osteoporosis compared with the patients without moderate/severe vertebral fracture (Table 1). Additionally, a higher proportion of women had vertebral fractures than men. The rates of diagnosed (ICD-coded) vertebral fractures and osteoporosis were generally low. Specifically, only 31% of patients with CT-verified vertebral fractures had an ICD-coded diagnosis of vertebral fracture, and 33% had a diagnosis of osteoporosis. Similarly, osteoporosis treatment was limited, with only 17% of individuals in the group with vertebral fracture undergoing treatment. However, compared to patients without previous vertebral fracture, the treatment rate was significantly higher in patients with previous vertebral fractures.

**Table 1:** Comparison of patients with and without vertebral fracture in CT scan

	All (*n* = 101)	No or mild VF (*n* = 53)	Moderate or severe VF (*n* = 48)	*p*-value comparing no/mild vs. moderate/ severe VF
Age (years)	84 ± 7	83 ± 8	85 ± 7	0.138
Sex (%Female)	42%	28%	56%	0.005
Previous fracture*	48%	30%	67%	< 0.001
Previous osteoporosis*	20%	8%	33%	0.002
Previous VF*	17%	4%	31%	< 0.001
Osteoporotic treatment^#^	10%	4%	17%	0.044
Calcium/Vitamin-D treatment	16%	8%	25%	0.027

Mean ± SD

*VF* vertebral fracture

*ICD-coded

^#^Current medical treatment according to patients’ digital case record 24 h before fall incident, except for zoledronic acid that was considered current if given within 1 year before the fall incident

### AI versus consensus—all

As shown in Table 2, the AI algorithm showed a high accuracy (0.93), sensitivity (0.86), and specificity (0.99), when looking at the whole cohort. Sensitivity was lower at individual vertebral analysis (0.71), but with similar levels of accuracy (0.96) and specificity (0.99).

**Table 2:** Performance of AI-algorithm compared to ground truth

	Subject level	Vertebra level
Number	246	2136
Accuracy	0.93 (0.89–0.96)	0.96 (CI 0.95–0.97)
Sensitivity	0.86 (0.78–0.92)	0.71 (CI 0.64–0.78)
Specificity	0.99 (0.96–1.00)	0.99 (CI 0.98–0.99)
PPV	0.98 (0.95–1.00)	0.82 (CI 0.76–0.88)
NPV	0.89 (0.84–0.94)	0.97 (CI 0.97–0.98)
Cohen’s Kappa	0.85 (0.78–0.91)	0.74 (CI 0.69–0.80)
AUC	0.92 (0.88–0.96)	0.85 (CI 0.81–0.89)

### AI versus consensus—subgroup analysis

Generally, all subgroup analyses showed good performance including accuracy with values varying from 0.88 to 0.96, specificity 0.96 to 1.00, and sensitivity 0.75 to 0.95 (Tables 3 and 4). Accuracy was higher in females than in male (0.96 vs. 0.89, *p* = 0.033) and in scans performed with non-bone kernels compared to bone kernels (0.96 vs. 0.88, *p* = 0.020). No significant difference was found regarding accuracy of different anatomic regions (lumbar vs. thoracic region), contrast phases (non-contrast vs. contrast scans), or age (< 85 vs. ≥ 85 years).

**Table 3:** Performance of AI-algorithm depending on sex and age

	Female	Male	Age < 85 years	Age ≥ 85 years
Numbers	114	132	125	121
Accuracy	0.96 (0.92–0.99)	0.89 (0.83–0.94)	0.90 (0.85–0.95)	0.95 (0.91–0.98)
Sensitivity	0.95 (0.89–1.00)	0.75 (0.63–0.87)	0.80 (0.70–0.90)	0.91 (0.83–0.98)
Specificity	0.98 (0.94–1.00)	0.99 (0.96–1.00)	0.99 (0.96–1.00)	0.98 (0.95–1.00)
PPV	0.98 (0.95–1.00)	0.97 (0.91–1.00)	0.98 (0.93–1.00)	0.98 (0.94–1.00)
NPV	0.95 (0.88–1.00)	0.86 (0.78–0.93)	0.86 (0.79–0.94)	0.93 (0.86–0.99)
Cohen’s Kappa	0.93 (0.84–0.98)	0.77 (0.64–0.87)	0.80 (0.69–0.90)	0.90 (0.81–0.97)
AUC	0.96 (0.93–1.00)	0.87 (0.79–0.94)	0.89 (0.83–0.95)	0.95 (0.90–0.98)

**Table 4:** Performance of AI-algorithm depending on kernel, anatomic region, and contrast phases

	Bone kernel	None bone kernel	Region thoracic	Region lumbar	Contrast enhanced CT	No contrast enhancement
Numbers	100	146	128	118	108	138
Accuracy	0.88 (0.81–0.94)	0.96 (0.92–0.99)	0.91 (0.85–0.95)	0.95 (0.91–0.98)	0.91(0.85–0.96)	0.94 (0.9–0.98)
Sensitivity	0.78 (0.66–0.89)	0.91 (0.84–0.97)	0.81 (0.70–0.91)	0.90 (0.81–0.97)	0.81 (0.70–0.91)	0.90 (0.81–0.97)
Specificity	0.96 (0.91–1.00)	1.00 (1.00–1.00)	0.97 (0.93–1.00)	1.00 (1.00–1.00)	1.00 (1.00–1.00)	0.97 (0.94–1.0)
PPV	0.95 (0.86–1.00)	1.00 (1.00–1.00)	0.96 (0.89–1.00)	1.00 (1.00–1.00)	1.00 (1.00–1.00)	0.96 (0.91–1.0)
NPV	0.84 (0.75–0.93)	0.93 (0.87–0.98)	0.88 (0.80–0.94)	0.91 (0.83–0.97)	0.85 (0.75–0.93)	0.93 (0.87–0.98)
Cohen’s Kappa	0.75 (0.62–0.88)	0.92 (0.84–0.97)	0.80 (0.69–0.90)	0.90 (0.81–0.97)	0.81 (0.70–0.92)	0.88 (0.79–0.96)
AUC	0.87 (0.80–0.93)	0.95 (0.92–0.99)	0.89 (0.83–0.95)	0.95 (0.90–1.00)	0.91 (0.84–0.97)	0.94 (0.89–0.99)

## Discussion

The present study of mixed CT scans with different scan protocols from the thorax and the abdomen showed a high performance of the tested AI tool to opportunistically detect vertebral fractures in a geriatric setting. A consistently high performance was seen across all studied subgroups (accuracy 0.88 to 0.96).

Our study cohort comprised high-risk individuals for vertebral fractures, characterized by risk factors such as advanced age (mean 84 years), a high number of previous fractures (48%), and at least one previous fall. Consequently, the prevalence of moderate to severe vertebral fractures was high (45%). Similarly, a previous study by van der Jagt-Willems et al. [22] on a geriatric cohort (mean age 82 years) reported a high prevalence of vertebral fractures (51% with mild to severe and 35% with moderate to severe vertebral fractures). Generally, in older cohorts, the prevalence of moderate to severe vertebral fractures is expected to be high.

Our results showed an AI performance with high accuracy (0.93), sensitivity (0.86), and specificity (0.99), resulting in a low risk of overdiagnosis, but an identification rate of nearly 9 out of 10 patients with vertebral fractures. A high prevalence of the condition in the study cohorts, i.e., VF in our case, could possibly affect the result. Thus, AI algorithms tend to have higher precision in detecting findings when there is a high prevalence of the condition, as the increased number of positive cases enhances the algorithm’s ability to accurately identify them. However, a recently published article by Nicolaes et al. showed similar results (accuracy 0.92, sensitivity 0.81, and specificity 0.95) when performing external validation of the same AI algorithm as ours but in a cohort with lower VF prevalence, i.e., 15% moderate or severe VF and lower age, mean age 68–74 [18]. Studies with other AI algorithms and slightly younger cohorts (62–73 years) and lower VF prevalence (8–25%) have shown varying performance including moderate sensitivity 0.65 and high specificity 0.92 [17], or high sensitivity (0.94 to 1.00) but lower specificity (0.65 to 0.35) [14, 16].

At present, less than one out of three vertebral fractures come to clinical awareness [10–12]. Similarly, in our study, we found that less than half (44%) of the present vertebral fractures were reported in the original CT radiology report, and only 31% of patients with present vertebral fractures were previously ICD-coded. A sensitivity of 0.86 using AI-supported detection in real-life data, as shown in the present study, could thus have considerable potential to improve the care in osteoporosis, especially if implemented appropriately in the clinical setting well aligned with a fracture liaison service, where the radiological finding is effectively processed and forwarded to the clinical osteoporosis unit for further investigation and treatment.

All subgroup analyses demonstrated high accuracy ranging from 0.88 to 0.96, with sensitivities ranging from 0.75 to 0.95 and specificities 0.96 to 1.00.

Regarding possible sex differences, though with generally high levels, the AI algorithm performed slightly better in females compared to males (accuracy 0.96 vs. 0.89). This discrepancy was unexpected but might be attributed to the algorithm being trained on a larger number of CT scans originating from female patients. Additionally, the higher prevalence of vertebral fractures among females in this dataset might have influenced the results as a higher number of positive cases might favor a higher detection rate. Previous studies on AI algorithms have, to the best of our knowledge, not reported statistical analysis of sex differences.

Non-bone kernel scans showed higher accuracy than bone kernel scans (0.96 vs. 0.88) in the present study. Bone kernels are used in CT protocols to improve bone visualization and other prominent structures with hard edges, whereas non-bone kernels are used to enhance soft tissue visualization (e.g., liver, kidneys, fat, and muscles) [23]. A harder edge visualization and more noise (Fig. 1B) could possibly affect the AI’s judgment of vertebral border and ability to identify fractures which might be one reason for the differences observed in our study. Non-bone kernel scans are more often used in general and thus possibly the most frequent scan for opportunistic screening. One possible reason for the observed difference in our study might be that the AI algorithm was mainly trained on data with softer, non-bone kernels.

Despite the theoretical diagnostic challenges posed by degenerative changes in an aging spine, our study found no significant difference regarding performance between the age-groups, i.e., over or under 85 years (accuracy 0.95 to 0.90). Most previous studies were done in younger patient cohorts, i.e., mean age 62–78 years [14, 16, 18], generally having spinal appearances with less degenerative changes. The present finding indicates a robustness of the AI algorithm regarding analysis of more radiologically challenging scans.

Presence of contrast agents increase the Hounsfield units which significantly alters the bone mineral estimation [16, 24]. Theoretically, this could also affect the AI-algorithm performance by differences in defining the vertebral borders and presence of vertebral fractures. In the present study, however, there were no differences regarding the performance between non-contrast and contrast enhanced CT scans (accuracy 0.91 to 0.94, sensitivity 0.81 to 0.90, and specificity 0.97 to 1.00). In agreement with our study, no difference in the detection rate between CT scans with or without contrast agent analyzed with HealthVCF, Zebra Medical was found in a previous study performed by Roux et al. [16].

Limitations of the present study include the fact that most CT scanners are from the same manufacturer, and the study was conducted at a single center, which may limit the generalizability of the data. Additionally, some patients had multiple CT scans included in the analysis. On the other hand, a strength of our study lies in the well-characterized CT protocol parameters within the dataset, as well as the investigation of patients using different protocols with various clinical indications.

Based on the performance and current experience with the AI results, a prospective study should be conducted. This should aim to integrate the algorithm in the daily workflow and optimize the interaction by medical users and the following patient journey. The integration of AI into existing clinical workflows necessitates significant changes in infrastructure, training for healthcare providers, and updates to clinical practice guidelines. It is crucial to keep the false positive rate of detected fractures to a minimum, to avoid increasing the workload for the radiologist. The full impact of AI in vertebral fracture detection on the treatment and outcome of osteoporosis patients has not been demonstrated yet due to lack of prospective studies.

## Conclusion

The present study shows high performance of AI-supported vertebral fracture detection, including subgroup analysis, in real-world opportunistic CT analysis of thoracic and abdominal scans in a geriatric cohort. Performance was better in females than in males and in non-bone kernel vs. bone kernel scans.

## Data Availability

The data underlying this article will be shared on reasonable request to the corresponding author.
